# Chemometric-Assisted Spectrophotometric Method for the Simultaneous Determination of Ciprofloxacin and Doxycycline Hyclate in Pharmaceutical Formulations

**DOI:** 10.1155/2018/9538435

**Published:** 2018-12-18

**Authors:** Tadele Eticha, Getu Kahsay, Fitsum Asefa, Teklebrhan Hailu, Hailekiros Gebretsadik, Tesfamichael Gebretsadikan, Boovizhikannan Thangabalan

**Affiliations:** School of Pharmacy, College of Health Sciences, Mekelle University, Mekelle, Ethiopia

## Abstract

Two chemometrics methods—principal component regression and partial least squares—were developed for simultaneous spectrophotometric estimation of ciprofloxacin and doxycycline hyclate in pharmaceutical dosage forms without any pretreatment. The UV spectra of both drugs were recorded at concentrations within their linear ranges between 200 and 400 nm with the intervals *λ* = 2 nm at 100 wavelengths in distilled water. Beer's law was obeyed for both drugs in the concentration ranges of 1–10 *μ*g/mL for ciprofloxacin and 5–25 *μ*g/mL for doxycycline hyclate. Two sets of standard mixtures, 25 as a calibration set and 9 as a validation set, were prepared. The calibration models were evaluated by cross-validation and external validation over synthetic mixtures. The optimized models were successfully applied for chemometric analysis of ciprofloxacin and doxycycline hyclate in synthetic and pharmaceutical mixtures with satisfactory accuracy (recovery values from 97.50% to 101.87%) and precision (RSD < 2%).

## 1. Introduction

Ciprofloxacin (CIP), chemically 1-cyclopropyl-6-fluoro-4-oxo-7-(piperazin-1-yl)-1,4-dihydroquinoline-3-carboxylic acid, belongs to the class of the fluoroquinolone antibiotics [1]. It is used to treat bacterial infections such as gastrointestinal, Intra-abdominal, skin, respiratory tract, urinary tract, and bone and joint infections, among others [2]. Doxycycline (DOX), chemically (4S,4aR,5S,5aR,6R,12aS)-4-(Dimethylamino)-3,5,10,12,12a-pentahydroxy-6-methyl-1,11-dioxo-1,4,4a,5,5a,6,11,12a-octahydrotetracene-2-carboxamide, is an antibiotic which belongs to the group of tetracyclines [1]. It is used to treat pelvic inflammatory disease, chronic prostatitis, sinusitis, acne, and rickettsial infections in addition to the general indications for all members of the tetracycline antibiotics group [2].

Both ciprofloxacin and doxycycline hyclate are official in the British and United States Pharmacopoeia, and their official method of assay is high-performance liquid chromatography (HPLC) [1, 3]. Several analytical methods such as spectrophotometry [4, 5], fluorimetry [6], ultra-performance liquid chromatographic [7], HPLC [8], high-performance thin-layer chromatography [9], high-performance capillary electrophoresis [10], and capillary electrophoresis [11] have been reported for the determination of ciprofloxacin while spectrophotometry [12, 13] and HPLC [14, 15] are for the determination of doxycycline hyclate alone or in combination with other medicines.

In recent years, the application of chemometrics, particularly multivariate calibration methods, is playing a crucial role in the multicomponent analysis of pharmaceutical mixtures [16]. Multivariate calibration methods such as PCR and PLS applied to spectral data are being increasingly used for instrumental methods without separation techniques [17, 18]. Multivariate calibration of PCR and PLS have been applied for simultaneous UV-Vis spectrophotometric determination of ciprofloxacin and ornidazole [19], ciprofloxacin and tinidazole [20], ambroxol and doxycycline [21], and *β*-lactam antibiotic binary mixtures [22] in pharmaceutical formulations.

The purpose of this study was to introduce alternative analytical procedures based on the chemometric-assisted spectrophotometric methods for the simultaneous determination of ciprofloxacin and doxycycline hyclate in laboratory-prepared synthetic and pharmaceutical mixtures. Using an apparatus at a time for the simultaneous determination of both drugs using water as a solvent is a great benefit for developing nations because of the efficient use of the instrument and cheap solvent. Therefore, these analytical methods might be helpful specifically in developing countries where resource is limited.

## 2. Experimental

### 2.1. Apparatus and Software

Spectrophotometric measurements were performed on a double-beam UV-VIS spectrometer (PG Instruments, Lutterworth, England), equipped with 1 cm matched quartz cells, connected to a computer loaded with UV-Win PC software. All absorption spectra were saved and subsequently exported UV-Win software to Microsoft Excel program for statistical manipulation. Minitab 7.1 version software was employed to determine concentrations of combinations for calibration and validation sets while Unscrambler® X 10.5 version was used for PCR and PLS model development and data analysis.

### 2.2. Reagents and Samples

Pharmaceutical grade ciprofloxacin and doxycycline were obtained from Addis Pharmaceutical Factory (Adigrat, Ethiopia). Distilled water was used throughout the work. Commercial samples, ciprofloxacin tablets labeled to contain 500 mg (Serviflox, Sandoz and Zindolin, Remedica) and doxycycline hyclate labeled to contain 100 mg capsules (Doxicad, Cadila), and tablets (Remycin, Remedica), were purchased from the local pharmacies in Mekelle, Ethiopia.

### 2.3. One-Component Calibration

This was examined in the concentration range of 1–10 *μ*g/mL for ciprofloxacin and 5–25 *μ*g/mL for doxycycline hyclate. Absorbance values were recorded at *λ*_max_ of each drug (272 nm for CIP and 275 nm for DOX) against distilled water as blank. Linear dynamic range for each drug was studied by least square linear regression of concentration and the corresponding absorbance.

### 2.4. Preparation of Stock and Working Standard Solutions

Accurately weighed and transferred (10 mg) either of the standard drugs into 100 mL volumetric flask was dissolved in about 80 mL of distilled water and diluted to volume with the same solution. Suitable aliquots of the stock solutions were diluted with the solvent to obtain the appropriate working standard solutions according to the linear calibration range for each drug.

### 2.5. Construction of Calibration and Validation Sets

The calibration and validation mixtures were prepared by combining working standard solutions of CIP and DOX in different ratios in their concentration linearity ranges. The concentrations of mixtures were determined by general factorial design (2 factors at 5 level of each factor) for calibration set and (2 factors at 3 level of each factor) for validation set. A total set of 25 and 9 calibration (Table 1) and validation (Table 2) mixtures were independently prepared, respectively. The absorption spectra of all mixtures were recorded over the range 200–400 nm with 2 nm interval.

### 2.6. Analysis of the Marketed Formulations

Twenty tablets or capsules were accurately weighed and finely powdered. Tablet or capsule powder equivalent to ciprofloxacin (100 mg) and doxycycline hyclate (150 mg) were accurately weighed and transferred into 100 mL volumetric flask, and 50 mL of distilled water was added. The solution was well shaken and ultrasonicated for 15 min. Then, the solution was filtrated in a 100 mL volumetric flask through a Whatman filter paper # 42 filter paper. The residue was washed three times with 10 mL water, and the volume was completed to 100 mL with water. Suitable aliquots of the stock solutions were mixed and diluted with the solvent to obtain the appropriate working sample solution for UV measurements at the specified range.

Synthetic mixture was used for the combination of working standard solutions while pharmaceutical mixture referred to the combination of sample solutions throughout this work.

### 2.7. Accuracy Study

The accuracy of the method was performed at three levels 80, 100, and 120% of the working concentration of sample. Calculated amounts of standard solution of CIP and DOX were spiked into sample solution, and the resulting solutions were scanned in the range of 200–400 nm. The accuracy of the method was evaluated as the percent recovery of the added amounts of the standard to the previously analyzed sample. The developed method was validated according to the International Conference on Harmonization (ICH) guidelines.

## 3. Results and Discussion

### 3.1. Multivariate Calibration Analysis

**Figure 1:**
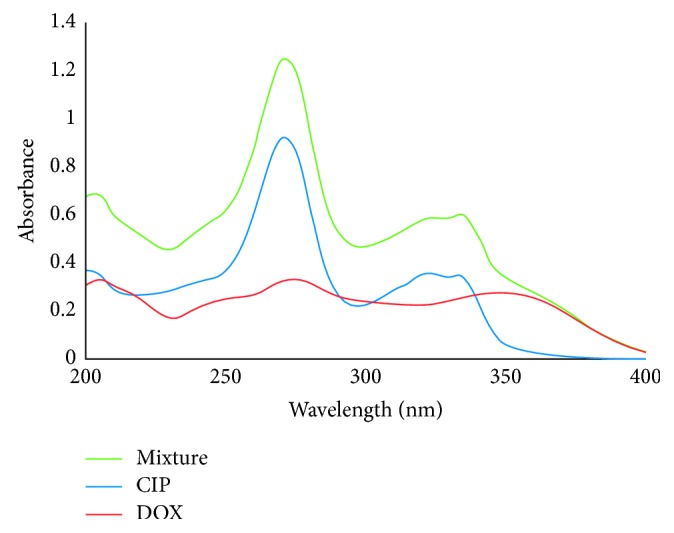
UV absorption spectra of ciprofloxacin, doxycycline hyclate, and the mixture.

The UV absorption spectra of ciprofloxacin, doxycycline hyclate, and their mixture are given in Figure 1. The calibration and prediction sets were designed in 25 and 9 laboratory-made mixtures (Tables 1 and 2) as described in Section 2.5. The absorbance spectra which showed a significant overlap were recorded between 200 and 400 nm in the intervals as Δ*λ* = 2 nm at 100 wavelengths. Multivariate calibrations such as PCR and PLS are therefore required for such analysis due to presence of interference.

A number of laboratory synthetic mixtures (Table 2) were subjected to the PCR and PLS analysis to prove the suitability of the calibration model for determination of ciprofloxacin and doxycycline hyclate in the pharmaceutical sample solutions. The model was built with the help of the Unscrambler software. The findings were satisfactory as the concentrations of each drug predicted by the model are close to the actual concentrations.

The predictive abilities of the models were evaluated by root-mean-square error of cross-validation (RMSECV), root-mean-square error of prediction (RMSEP), and correlation coefficient (*r*^2^) (Table 2). With cross-validation, the same calibration set samples were used for both model estimation and testing. Leave-one-out cross-validation, leaving out one sample at a time, was employed to validate the PCR and PLS models in model development. The models were also validated by prediction of the concentration of analytes in separate validation set which was not used in the model development (Table 2). The RMSEP generated from validation set is the estimated prediction error that accurately reflects all sources of variability in the calibration method. To validate the model, both RMSECV and RMSEP must be as low as possible for a model [23]. Reasonable correlation coefficient was obtained for each drug in the validation set samples by PCR- and PLS-optimized models, demonstrating good predictive abilities of the models.

### 3.2. Accuracy

The reliability and validity of the proposed method were examined by the standard addition technique at 80%, 100%, and 120% of the test concentration. The mixtures were analyzed, and the percent recoveries ranged from 97.50% to 101.87% (Table 3). These findings confirmed that the excipients in pharmaceutical products do not interfere with the determination of ciprofloxacin and doxycycline hyclate.

Precision of the developed method was examined as intra- and interday precisions. Six determinations of the drug solution were performed for three consecutive days for the evaluation of intermediate precision. The percent relative standard deviations of both precisions were <2%, revealing the good precision of the method.

### 3.3. Analysis of Real Samples

The proposed methods were applied for the assay of ciprofloxacin and doxycycline hyclate in laboratory prepared mixtures of their pharmaceutical preparations. The assay results are shown in Table 4. The findings were found to be in a good agreement with the concentration taken for the formulations. This revealed that the matrices and/or excipients did not interfere with the quantifications.

The developed techniques are much easier than HPLC methods stipulated in pharmacopeias [1, 3] and other existing analytical methods [7, 8, 14, 15]. The methods employed distilled water as a solvent, and the procedures do not involve any sample pretreatment.

## 4. Conclusion

The developed UV spectrophotometric methods in combination with PCR and PLS can be used for the simultaneous determination of ciprofloxacin and doxycycline hyclate in laboratory-prepared binary mixtures of their either pure powder forms or pharmaceutical preparations. The proposed techniques do not need any sample pretreatment, and they are rapid, precise, and accurate. Therefore, these methods could be suitable for quality-control laboratories especially in developing nations, where resource is limited.

## Figures and Tables

**Table 1:** Composition of calibration set.

Mix. no.	CIP (*μ*g/mL)	DOX (*μ*g/mL)
1	1	15
2	2	7
3	4	9
4	1	7
5	2	5
6	4	5
7	6	5
8	6	12
9	8	7
10	4	15
11	8	5
12	4	12
13	4	7
14	1	12
15	8	15
16	6	15
17	1	5
18	8	12
19	2	9
20	1	9
21	2	15
22	2	12
23	6	9
24	8	9
25	6	7

**Table 2:** Composition of validation set and predicted results obtained in synthetic mixtures by PCR and PLS methods.

CIP	DOX	PCR				PLS			
		CIP		DOX		CIP		DOX	
Actual conc. (*μ*g/mL)		Predicted (*μ*g/mL)	% recovery	Predicted (*μ*g/mL)	% recovery	Predicted (*μ*g/mL)	% recovery	Predicted (*μ*g/mL)	% recovery
5	13	4.99	99.72	12.96	99.69	4.99	99.73	12.96	99.66
7	10	7.04	100.60	10.00	100.00	7.04	100.61	10.01	100.05
5	7	5.09	101.75	7.00	100.00	5.09	101.76	7.00	100.00
7	7	7.18	102.60	7.09	101.29	7.18	102.61	7.09	101.30
7	13	7.03	100.50	13.23	101.77	7.04	100.52	13.23	101.79
5	10	4.95	98.95	9.90	99.00	4.95	98.96	9.90	99.00
3	7	2.72	90.52	6.68	95.43	2.72	90.55	6.68	95.40
3	13	2.88	95.98	12.70	97.69	2.88	96.00	12.70	97.66
3	10	2.79	92.88	9.65	96.50	2.79	92.89	9.65	96.49
*R* ^2^		0.998		0.994		0.998		0.994	
Intercept		0.376		0.210		0.379		0.210	
Slope		0.932		0.988		0.931		0.988	
RMSECV		0.278		0.794		0.283		0.824	
RMSEP		0.142		0.219		0.143		0.208	

*R* ^2^, correlation coefficient; RMSECV, root-mean-square error of cross-validation; RMSEP, root-mean-square error of prediction.

**Table 3:** Accuracy data of CIP and DOX by PCR and PLS models (*n*=3).

Mix.	Component	Level (%)	Amount taken (*μ*g/mL)	PCR			PLS		
				Predicted (*μ*g/mL)	% recovery	% RSD	Predicted (*μ*g/mL)	% recovery	% RSD
Mix. 1	CIP tablet	80	3.2	3.12	97.50	0.524	3.13	97.81	0.662
		100	4	3.91	97.75	0.869	3.90	97.50	0.794
		120	4.8	4.83	100.63	0.979	4.82	100.42	1.021
	DOX capsule	80	8	17.71	98.39	0.790	17.71	98.39	0.783
		100	10	20.23	101.15	0.941	20.24	101.20	0.933
		120	12	21.73	98.77	0.834	21.74	98.82	0.829

Mix. 2	CIP tablet	80	3.2	3.26	101.87	1.261	3.24	101.25	1.112
		100	4	3.93	98.25	0.634	3.92	98.00	0.751
		120	4.8	4.79	99.79	0.740	4.78	99.58	0.641
	DOX tablet	80	8	17.82	99.00	1.111	17.83	99.06	1.101
		100	10	20.19	100.95	0.799	20.2	101.00	0.791
		120	12	21.77	98.95	0.372	21.79	99.05	0.368

**Table 4:** Assay results of CIP and DOX in laboratory-prepared commercial mixtures by developed PCR and PLS methods (*n*=3).

Mix.	Dosage form	Component	Actual conc. (*μ*g/mL)	PCR		PLS	
				Predicted (*μ*g/mL ± SD)	% recovery	Predicted (*μ*g/mL ± SD)	% recovery
Mix. 1	Tablet	CIP	8	7.94 ± 0.064	99.19	7.94 ± 0.065	99.25
	Capsule	DOX	12	12.13 ± 0.548	101.08	12.13 ± 0.560	101.08

Mix 2	Tablet	CIP	8	7.90 ± 0.039	98.75	7.91 ± 0.041	98.81
	Tablet	DOX	12	11.74 ± 0.974	97.79	11.74 ± 0.041	97.79

## Data Availability

The data used to support the findings of this study are available from the corresponding author upon request.
